# Influence of the preparation design on the retentive strength of resin-bonded attachments

**DOI:** 10.1007/s00784-024-06017-2

**Published:** 2024-11-05

**Authors:** Jorge Wasmund, Sebastian Wille, Matthias Kern

**Affiliations:** Department of Prosthodontics, Propaedeutics and Dental Materials, School of Dentistry, Christian-Albrechts University at Kiel, Arnold-Heller-Straße 16, 24105 Kiel, Germany

**Keywords:** Resin-bonded attachments, Removable dental prosthesis, Retention grooves, Retainer wing thickness

## Abstract

**Objectives:**

The study investigated the influence of retention grooves and material thickness of the retainer wing on the retentive strength of resin-bonded attachments (RBAs).

**Materials and methods:**

Sixty-four extracted human molars were used. Each tooth received a preparation limited to the enamel for the retainer wings of the RBAs. The specimens were divided into eight groups, each containing eight specimens. The groups varied based on the number of conical retention grooves (0, 1, 2, or 4) and the thickness of the CoCr retainer wings (0.4 mm and 0.8 mm). Before testing the retentive strength of the RBAs, the specimens underwent 37,500 thermal cycles followed by dynamic loading of 1,200,000 cycles on the RBAs’ patrices using a chewing simulator. The debonding test was conducted using a universal testing machine at a crosshead speed of 2 mm/min.

**Results:**

The mean retentive strength ranged from 326 ± 96 N to 440 ± 77 N. Only a small portion of specimens (10.9%) exhibited adhesive failure, while the remainder demonstrated cohesive failure within the tooth structure. Neither the number of retention grooves, the thickness of the retainer wings, nor the size of the bonding surface significantly affected retentive strength.

**Conclusions:**

The findings suggest that reducing the number of retention grooves and the material thickness does not influence the retentive strength of RBAs.

**Clinical relevance:**

The retention of RBAs appears promising, supporting the clinical application of this treatment modality.

## Introduction

Minimally invasive restorations are increasingly favored by many patients due to the reduced loss of tooth structure. Unlike conventional crown preparations, which can result in up to a 75% loss of coronal tooth structure, minimally invasive preparation keeps this loss to a minimum [1, 2]. This approach also minimizes the risk of pulp damage. Because these preparations are limited to the enamel and performed with sufficient water cooling, the temperature in the pulp cavity remains below critical levels [3]. A rise in temperature of 5.5 °C in the pulp chamber can cause pulp necrosis in 15% of teeth [4].

Resin-bonded attachments (RBAs) are a minimally invasive option for retaining removable partial dentures (RPDs), incorporating additional coronal attachments connected to retainer wings [5]. RBAs share a clinical workflow with resin-bonded fixed partial dentures (RBFDPs), as both involve similar preparation steps, including lingual veneer preparation, shallow cervical beveling, parallel retention grooves, and occlusal rests [6]. Retentive features such as grooves and holes can improve the success of resin-bonded restorations by enhancing retention strength [7, 8]. For RBAs, two parallel retention grooves, prepared on the mesial and distal surfaces of the tooth, are recommended [6, 9].

Another factor influencing the retentive strength of RBAs is the thickness of the metal retainer wing. While studies have shown that increased thickness improves retention strength in RBFDPs, no such studies exist for RBAs. A minimum thickness of 0.5 mm is recommended for RBFDPs [6], with additional research confirming that thicker materials enhance retention [10–13].

However, a recent laboratory study on premolars suggests that reducing the material thickness might be justified. Likewise, reducing the number of retention grooves could be beneficial [14]. To ensure secure and precise positioning of RBAs during bonding, it is advised to apply two retention grooves under rubber dam isolation [14]. The retentive strength of RBAs could also be influenced by the speed of preparation, as high-speed enamel preparation may negatively affect retention [15]. Therefore, reduced rotation speeds are often recommended for minimally invasive enamel preparation [16]. However, a previous study has used a high-speed air turbine operating at 250,000 to 320,000 rpm [14], which may have adversely affected the superficial enamel structure.

Debonding is the most frequent cause of RBA failure [17]. Various studies have reported debonding rates ranging from 6 to 16%, depending on the observation period [17–20]. The average follow-up time was 1 to 7 years, with a maximum of 22 years. Over 80% of RBAs remained functional after 5 years, and 61% were functional after 15 years [9, 17].

In the past, prefabricated plastic attachments were predominantly produced using cast non-precious metals (NPM). However, advancements in Computer-Aided Design (CAD) and Computer-Aided Manufacturing (CAM) now allow for the milling of workpieces from zirconia blocks based on digital designs. Monolithic zirconia ceramics, recognized for their strength and durability, are widely applied in prosthodontics [21–24]. Nevertheless, metal remains the material of choice for RBAs, even with the development of ceramic alternatives. This preference is attributed to metals’ superior mechanical properties, particularly their greater ductility and resistance to dynamic forces, which reduce the risk of fractures. Therefore, zirconia RBAs require larger dimensions and are limited in terms of available space [29].

Given the existing scientific uncertainty regarding the influence of retention grooves and material thickness on RBAs, this study aimed to evaluate their effects on the retentive strength of RBAs.

The study specifically addressed the following questions:

1. Does reducing the material thickness from 0.8 mm to 0.4 mm decrease the retentive strength of RBAs?
2. Does the number of retention grooves affect the retentive strength of RBAs?

## Materials and methods

After informed consent had been obtained according to the regulations of the local ethical committee (number of the ethics vote: D528/19), sixty-four human, freshly extracted wisdom teeth are stored in a 0.1% thymol solution. Before being used for the experiments, the teeth were stored in distilled water at 5 °C for two weeks. The teeth were also cleaned with pumice powder (Ernst Hinrichs Dental, Goslar, Germany) and a nylon polishing brush (Polishing Brush Nylon 116; Hagar & Meisinger, Neuss, Germany) using a green contra-angle handpiece with a 4:1 gear ratio (29 A; KaVo, Biberach, Germany) at 1,500 rpm.

First, a horizontal canal, 1 mm in thickness, was drilled into the center of the tooth root in the buccal-lingual direction, maintaining a constant distance from the enamel-cement interface. A 1 mm thick metal wire was inserted into the tooth to enhance retention. To simulate physiological tooth mobility, an artificial tooth holding apparatus was created [25]. A liquid rubber (Liquid Tape; Plasti-Dip, Schwerin, Germany) was mixed with a universal thinner (Nitroverdünnung; FHG, Münster, Germany) in a 1:1 mass ratio until a liquid, homogeneous mixture was achieved. The sample teeth were then immersed for 5 s up to the enamel-cement interface. The tooth with the artificial periodontium was air-dried for 30 min until the mixture solidified.

A circular retention groove was created on the inside of the metal sleeves to improve the retention of the embedding material against extrusion. The tooth was fixed in the metal sleeve using cold-polymerizing polyester-based resin (Technovit 4000; Kulzer Technik, Wehrheim, Germany), with curing occurring at room temperature for one hour. The specimens were subsequently stored in distilled water at 5 °C. Each group comprised eight specimens. Four groups were created, each with material thicknesses of 0.4–0.8 mm. Within these groups, the number of retention grooves was varied (0, 1, 2, 4). The group codes, which correspond to the number of retention grooves and material thickness, are listed in Table 1.

Preparation was conducted using a red contra-angle handpiece (24B; KaVo, Bensheim, Germany) with a gear ratio of 1:4 and an instrument speed of 30,000 rpm, accompanied by a water-cooling rate of 50 ml/min. The procedure commenced in the mesial region of the tooth, progressing across the lingual side to the distal region, with a preparation height of 3.5 mm. A conical torpedo diamond bur (18878 K.014; Komet, Lemgo, Germany) was utilized for this process, creating a slight cervical bevel at the preparation margin.

In the sample groups with a material thickness of 0.8 mm, the amount of substance removal was intentionally limited to avoid involving the dentin. This resulted in a small overhang. By incorporating a convex design, the risk of food impaction is minimized. Furthermore, the preparation marginwas strategically placed in an area that is easily accessible for patients to maintain proper oral hygiene. Otherwise, achieving adhesion exclusively in the enamel would be challenging with a deeper preparation and no overhangs, potentially leading to significantly reduced adhesion values.

**Fig. 1:**
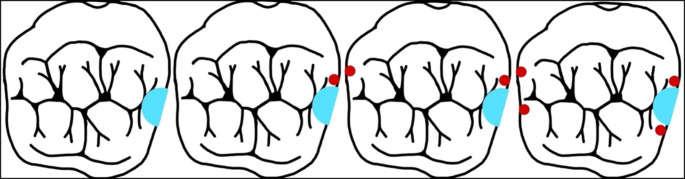
Localization of the retention grooves; from left to right 0G, 1G, 2G, 4G; the light blue area indicates the extent of the occlusal rest; the red dots mark the retention grooves

Depending on the specimen group, a specific number of retention grooves were created using a fine diamond separator (8850.012; Komet, Lemgo, Germany), each measuring 2.5 mm in length and 0.5 mm in depth. For multiple retention grooves, a minimum distance of 2 mm was maintained between grooves to prevent weakening of the tooth structure (see Fig. 1).

**Table 1:** Group codes of test groups

Number of retention grooves (G)	Material thickness		Number of specimens (*N* = 64)
	0.4 mm	0.8 mm	
0	(0G – 0.4)	(0G – 0.8)	16
1	(1G – 0.4)	(1G – 0.8)	16
2	(2G – 0.4)	(2G – 0.8)	16
4	(4G – 0.4)	(4G – 0.8)	16

**Fig. 2:**
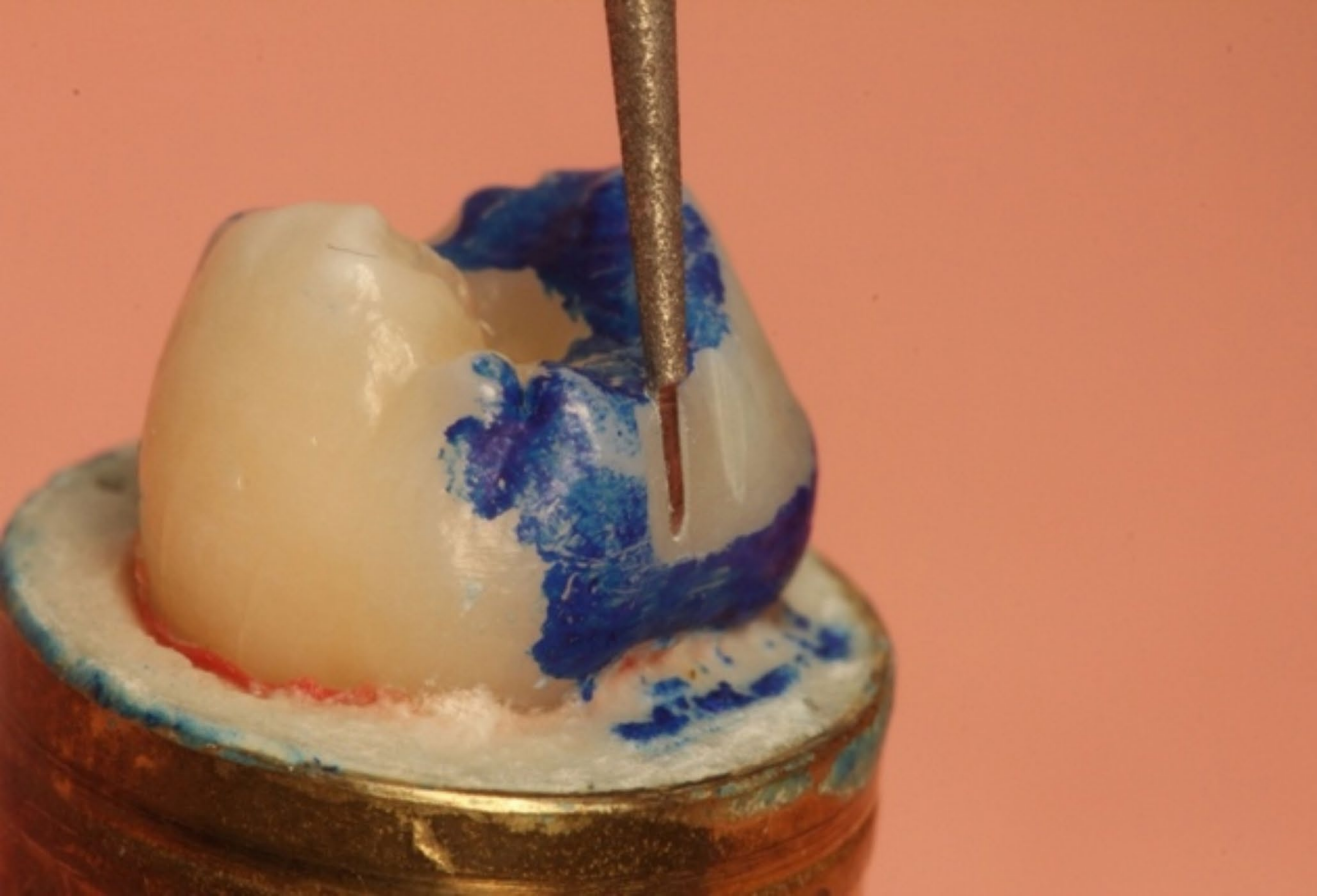
Checking the retention groove with the self-sharpened separating needle

An occlusal rest was prepared using a fine ball diamond (8801.018; Komet, Lemgo, Germany). The retention grooves were verified with a diamond-cut separating needle (8850.012; Komet, Lemgo, Germany) (see Fig. 2).

Impressions of the specimens were taken for laboratory work (Deguform; Densply-Sirona, Bensheim, Germany) to produce an embedding model (Optivest; Densply-Sirona, Bensheim, Germany) and a working model. The size of the prepared bonding surface in mm² was determined using a wax plate (smooth cast wax 0.4 mm; Bego, Bremen, Germany) and graph paper. All specimens were categorized into six groups based on the size of the bonding surface (see Table 2).

**Table 2:** Distribution of specimens by bonding surface

Groups	Bonding surface in mm^2^
1	< 70
2	70–74.5
3	75–79.5
4	80–84.5
5	85–89.5
6	≥ 90

The attachments were modeled on the previously fabricated embedding models using dental wax (Thowax cervical wax; Yeti, Engen, Germany), wax plates (0.4 mm and 0.8 mm), and semi-precision patrices (Preci-Vertix; Ceka, Hanover, Germany). The attachments were cast from a CoCr alloy (Wironit; Bego, Bremen, Germany).

(see Fig. 3).

**Fig. 3:**
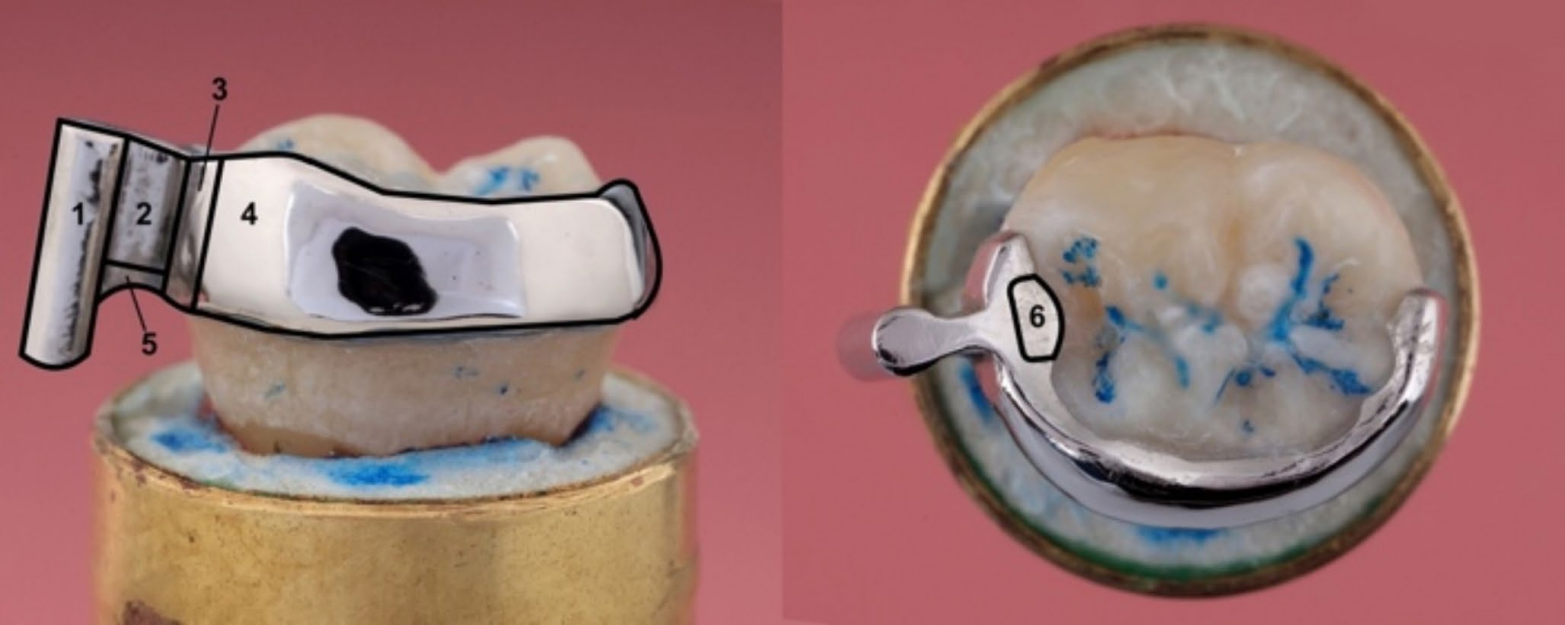
Lingual (left) and occlusal (right) view of a resin-bonded attachment, 1 = guide pin, 2 = connecting bar, 3 = stop plate, 4 = retainer wing, 5 = attachment point for the removal wire, 6 = occlusal rest

Casting occurred in an induction melting furnace (Nautilus, Bego, Bremen, Germany) at a temperature of 1460 °C. The cast RBAs were removed after the mold and casting object cooled passively to room temperature. Residual investment material was blasted off using an air blasting unit (Reco, Wiesbaden, Germany). The air blasting device employed 150 μm alumina particles at a pressure of 5 bar. The entire object was then blasted with a second air blasting unit (P-G 400; Harnisch und Rieth, Winterbach, Germany) using 50 μm alumina particles at a pressure of 2 bar. After detaching the cast objects, the attachments were placed on the working models. The RBAs were cleaned for three minutes in an ultrasonic bath (S30H Elmasonic; Elma, Singen, Germany) using 99% isopropanol.

**Fig. 4:**
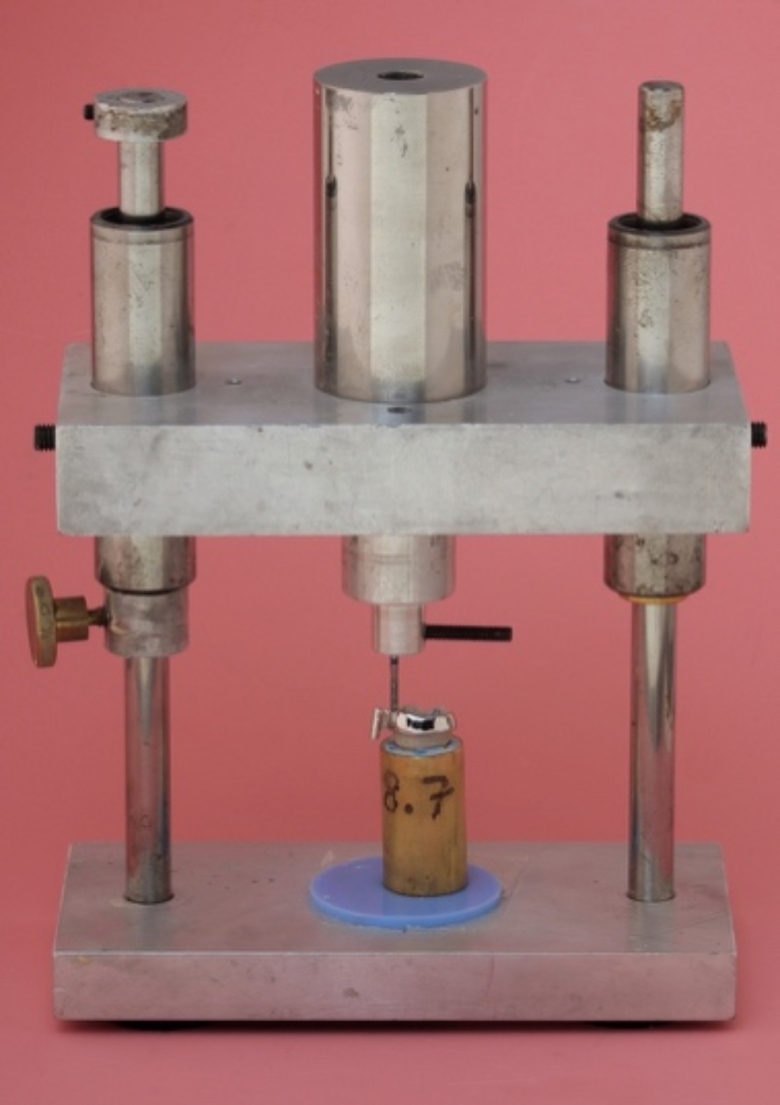
Positioning device with a load of 750 g

The auto-curing luting resin (Panavia 21 EX; Kuraray, Osaka, Japan), containing a phosphate monomer (10-methacryloyloxydecyl dihydrogen phosphate), was used to bond the RBAs to the etched enamel (etched for 30 s with 37% phosphoric acid; Pluraetch+; Pluradent, Grünwald, Germany). Finally, the enamel was rinsed with water spray for 30 s to remove any deposits. The tooth was then dried with compressed air for 15 s. A positioning device weighing 750 g was used to maintain constant pressure on the RBAs during cementation (see Fig. 4). The positioner was placed in a heating cabinet (Incu Line) at a temperature of 37 °C for 10 min to simulate intraoral temperatures during the curing of the self-curing luting resin.

**Fig. 5:**
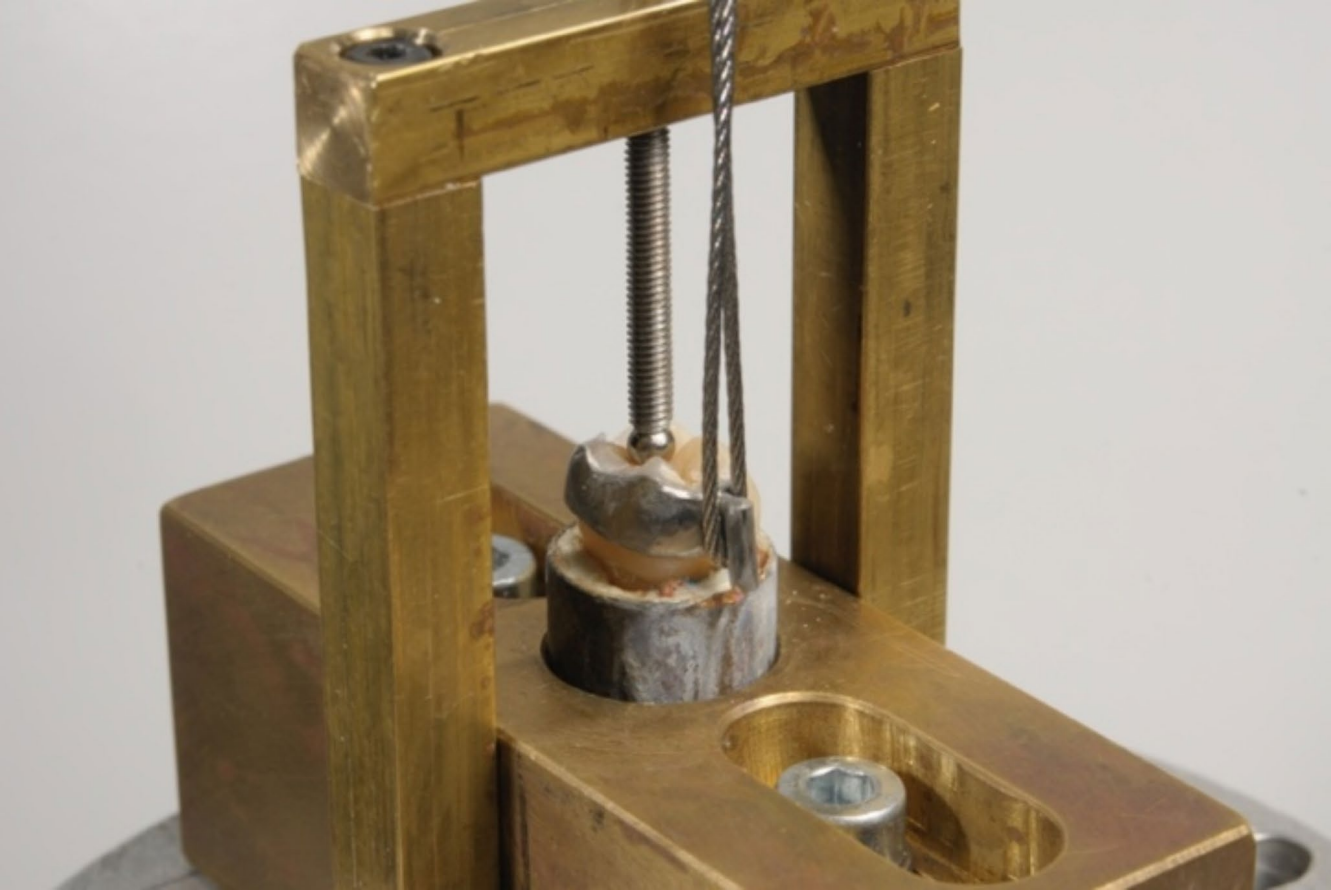
Specimen inserted in the universal testing machine

The specimens were stored in a 37 °C water bath (Precision GP02; Thermo Fisher Scientific, Newington, USA) for 150 days. During this period, the specimens were subjected to 37,500 temperature cycles (5 –55 °C). The specimens were then dynamically loaded on the patrices using a chewing simulator (CS4; SD-Mechatronik, Feldkirchen-Westerham, Germany) with a load of 5 kg at a frequency of 1.3 Hz and a loading speed of 30 mm/s for 1,200,000 loading cycles [26]. The retentive strength was evaluated using a universal testing machine (Z010; Zwick, Ulm, Germany) at a crosshead speed of 2 mm/min. All specimens that survived the chewing simulator without damage were placed in the universal testing machine (see Fig. 5).

A wire loop with a material thickness of 0.9 mm was utilized for the final test, which was inserted into a groove milled in front of the patrix.

The retentive strength was documented and statistically analyzed. A normal distribution could not be established for the test groups (Shapiro-Wilk test), and homogeneity of variance was not rejected (Levene test; *p* = 0.618). Consequently, the Kruskal-Wallis test was employed to analyze statistically significant differences between the groups. Additionally, the specimens were categorized into six new groups based on their available bonding surface, regardless of the number of retention grooves and material thickness. The Shapiro-Wilk test for the data of these groups did not reject the assumption of normal distribution (*p* > 0.05), and the Levene test also did not reject the homogeneity of variance (*p* = 0.180). Therefore, a one-way ANOVA was conducted to analyze the influence of the available bonding surface on retentive strength.

After debonding, the fracture types of the specimens were visually assessed as follows:

I = Detachment of the attachment with the tooth intact (debonding).

II = Enamel fracture.

III = Enamel-dentin fracture.

## Results

No specimens failed during thermal cycling and chewing simulation. Consequently, all specimens were evaluated for their retentive strength using the universal testing machine. Table 3 presents a descriptive analysis of the size of the bonding surface.

**Table 3:** Mean, standard deviation (SD), minimum (Min), and maximum values (Max) in mm² of the size of the bonding surface for each specimen group

Group	Mean	SD	Min	Max
0G – 0.4	80	9	70	93
1G – 0.4	81	8	70	96
2G – 0.4	77	10	66	92
4G – 0.4	77	7	66	86
0G – 0.8	73	6	66	82
1G – 0.8	76	7	70	85
2G – 0.8	71	4	65	77
4G – 0.8	77	7	66	87
All groups	77	7	65	96

Table 4 shows a descriptive table with the results of the debonding test. No statistically significant difference was found between the specimen groups (*p* = 0.118).

**Table 4:** Median, mean, standard deviation (SD), minimum (Min), and maximum values (Max) of the retentive strength in N from the debonding test for each group

Group	Median	Mean	SD	Min	Max
0G – 0.4	443	440	77	297	523
1G – 0.4	416	418	75	267	508
2G – 0.4	319	333	45	279	411
4G – 0.4	355	360	88	242	469
0G – 0.8	351	326	96	115	437
1G – 0.8	363	359	72	269	482
2G – 0.8	370	370	100	205	511
4G – 0.8	341	367	117	204	562
All groups	368	372	90	115	562

The retentive strength of the six groups (1–6) based on the available bonding surface, independent of the number of retention grooves and material thickness, is shown in Table 5. One-way ANOVA indicated no statistically significant differences (*p* = 0.262).

**Table 5:** Number of specimens (N), mean value, and standard deviation (SD) of the retentive strength within the bonding surface intervals from the debonding test

Group	Bonding surface in mm^2^	*N*	Mean	SD
1	< 70	12	328	65
2	70–74.5	19	371	97
3	75–79.5	11	374	79
4	80–84.5	12	380	72
5	85–89.5	7	392	128
6	≥ 90	3	464	89

Table 6 summarizes the fracture types descriptively without reference to groups. Debonding occurred in 10.9% of cases (fracture type I), while fractures involving the tooth structure occurred in 89.1% of cases (fracture types II and III).

**Table 6:** Number of specimens (N), percentage distribution (%), median, mean, standard deviation (SD), minimum (Min), and maximum (Max) values of the retentive strength for the three different fracture types (I = debonding, II = enamel fracture, III = enamel-dentin fracture)

Fracture types	*N*	%	Median	Mean	SD	Min	Max
I	7	10.9	275	339	136	204	562
II	29	45.3	353	359	74	242	516
III	28	43.8	400	393	89	115	523

## Discussion

Resin-bonded attachments (RBAs) provide a minimally invasive, esthetically pleasing, and durable solution for retaining removable dentures [1, 6, 27]. However, the widespread adoption of this method has been hindered by complex clinical procedures and insufficient training in prosthetic departments at dental schools [28]. The specimens in this study were prepared following clinically proven procedures, adhering to standards established in previous studies on RBAs [8, 14, 25, 29–31].

Minimally invasive preparation techniques help reduce the risk of dentin involvement, as verified by the color removal observed on the teeth. This approach significantly diminishes the weakening of tooth structure [1]. Furthermore, bonding to enamel yields higher and more durable bond strengths [32].

Preparation techniques also influence the roughness and surface structure of enamel, which depend on both the preparation speed and the diamond milling cutter grain size employed [15, 33]. To minimize damage to the enamel’s surface structure, a preparation speed of 30,000 rpm was utilized, aiming to reduce the occurrence of enamel cracks. Previous studies employing higher cutting speeds reported numerous fractures within the enamel, while the current study observed a significant reduction in enamel fractures and an increase in enamel-dentin fractures. This may be attributed to the reduced rotational speed, which appears to enhance the structural integrity of enamel and positively affect retentive strength.

All groups achieved sufficient attachment strength to the tooth, with retention tests indicating that the tooth structure failed in 89.1% of specimens, while bonding failure occurred in only 10.9%. The primary limitation on the retentive strength of specimens was the strength of the tooth structure. Since tooth structure failure predominated, no significant differences between groups were detected.

Surprisingly, the number of retention grooves did not influence the retentive strength. This finding may be explained by the low incidence of adhesive failure. It is posited that retention grooves serve to protect the adhesive layer of RBAs from peeling forces, thereby improving retentive strength in clinical applications. However, the tooth structure of the majority of the specimens fractured due to the superior adhesive strength of the MDP-containing luting resin, which obviously exceeded the cohesive strength of the enamel. Consequently, the influence of retention grooves on retention could not be established in this study. The high incidence of tooth structure fractures aligns with findings from previous research on metal and zirconia RBAs, which noted that human abutment teeth frequently fail following artificial aging and retentive strength testing [29]. The intrinsic strength of the teeth proved to be lower than the retentive strength of the RBAs. Thus, the chosen test configuration primarily evaluated the intrinsic strength of the teeth. The retention grooves were positioned along the tensile direction, without enhancing retention in this particular tensile orientation. They may be more critical in the presence of shear forces, where they could provide a mechanical effect in addition to increasing the adhesive surface and facilitating the transfer of rotational forces into the tooth.

Material thickness also did not significantly impact the retentive strength of RBAs, nor was there a notable difference in the type of fractures observed. A similar study found no positive influence of material thickness on retentive strength [14]. These results contradict earlier assumptions regarding the benefits of greater material thickness for resin-bonded fixed dental prostheses (RBFDPs) [6, 11–13], while simultaneously supporting findings from another study examining the influence of material thickness on the retentive strength of RBAs [14].

Interestingly, the size of the bonding surface also exhibited no significant effect on the retentive strength of specimens. This can be attributed to the cohesive strength of the tooth structure being the weak link in teeth restored with RBAs, rather than the bond strength of the luting resin.

Further investigation into the retentive behavior of RBAs is warranted, as the current test design only partially demonstrated the influence of retention grooves on retention. Compression and shear forces were not evaluated in the debonding test but may affect the clinical outcomes of RBAs.

## Conclusions

Reducing the material thickness of RBAs appears feasible, as no significant differences were observed between the two tested thicknesses. The bond strength between enamel and metal retainers of RBAs exceeds the cohesive strength of tooth substance and therefore does not appear to be a limiting factor in the success of RBAs.

## Data Availability

The authors declare that the data supporting the findings of this study are available within the paper . Should any raw data files be needed they are available from the corresponding author upon reasonable request.
